# When chronic critical illness is a family affair: A multi-perspective qualitative study of family involvement in long-term care

**DOI:** 10.1177/17423953221141134

**Published:** 2022-11-25

**Authors:** A Fuchsia Howard, Sarah Crowe, Laura Choroszewski, Joe Kovatch, Mary Kelly, Gregory Haljan

**Affiliations:** 1 School of Nursing, 8166 The University of British Columbia, Vancouver, British Columbia, Canada; 2 Department of Critical Care, Fraser Health Authority, Surrey, British Columbia, Canada; 3 Faculty of Medicine, 8166 The University of British Columbia, Vancouver, British Columbia, Canada

**Keywords:** Caregiver, family, critical illness, chronic illness, prolonged mechanical ventilation

## Abstract

**Objective:**

Those with chronic critical illness (CCI) remain dependent on life-sustaining treatments and increasingly reside in long-term care facilities equipped to meet their needs. The nature of family involvement in care remains undetermined thwarting approaches to mitigate poor family outcomes. The research objective was to explicate family involvement in the care of an individual with CCI who resides in long-term care.

**Methods:**

In this qualitative research, we used thematic analysis and constant comparative techniques to analyze data from interviews with 38 participants: 11 family members, 6 residents with CCI, and 21 healthcare providers.

**Results:**

Involvement in care entailed family: (1) reorienting their life despite the stress and emotional toll; (2) assuming responsibility for meaningful activities and management of practical matters, yet struggling alone; (3) advocating for care by being present, reminding and pushing, and picking their battles; and (4) figuring out how to contribute to nursing care, but with unclear expectations.

**Discussion:**

The burden of family caregiving was substantial, contrasting the assumption that family are relieved of their caregiver responsibilities when the patient with CCI is in a care facility. Research to address unmet family needs specific to their roles and responsibilities could potentially improve family outcomes and is warranted.

## Introduction

Roughly 80% of critically ill adults survive the acute phase in the intensive care unit (ICU), but 5% to 10% transition to persistent or chronic critical illness (CCI).^1^ Those with CCI experience persistent multi-organ dysfunction and remain dependent on life-sustaining treatments, primarily prolonged mechanical ventilation.^2,3^ They suffer a high symptom burden and profound physical, neurocognitive, and psychological impairments.^2,4^ Prognosis is poor with <20% of patients with CCI discharged home and a mortality rate of 50% at 1 year.^5–7^ Historically, these patients remained in an acute care hospital. However, various approaches and models of care have emerged to meet the demands of this growing population, including specific care programs in an ICU,^8,9^ respiratory care units,^10^ nurse-led special care units,^11^ specialized weaning centers,^12^ and long-term acute care hospitals.^13^ Patients with CCI are also increasingly discharged from hospital to a long-term care facility equipped to meet their complex care needs.

Family members of ICU survivors are at risk for long-term adverse psychological outcomes known as post-intensive care syndrome family, inclusive of anxiety, depression, symptoms of post-traumatic stress, loss of employment, and lifestyle interference.^14–16^ Emerging research suggests similar risks for family members of those with CCI, with depression found in half of the family members at hospital discharge,^17,18^ and one-third at 6-month post-hospital discharge.^18,19^ Symptoms of post-traumatic stress have been found in 16% to 18% of family members of patients with CCI, and associated with diminished health-related quality of life.^20,21^ Family have reported feeling worried by the chronically critically ill patient's unpredictable health and care transitions, unmet information and support needs, and disruptions to their lifestyle, employment, and financial stability.^12,19,22,23^ Surrogate decision makers can also experience distress, difficulty coping, decisional conflict and regret, and emotional and physical exhaustion.^24–27^

While the role of surrogate decision maker has been examined, family involvement in the care of a patient with CCI extends beyond this role. Moreover, the involvement of family members is likely context bound, such that where the individual with CCI resides (i.e. acute care hospital, weaning center, long-term care, or home) influences family experiences. In this study, we used a qualitative approach to explicate family involvement in the care of an individual with CCI who resides in long-term care, from the perspectives of family members, residents with CCI, and healthcare providers (HCPs). Long-term care facilities provide living accommodation for people who require on-site delivery of 24 h, 7 days a week supervised care, including professional health services, and personal care.^28^ These are often called nursing homes, personal care facilities, or residential continuing care facilities. In illuminating family involvement, our aim is to inform and improve approaches to supporting family members of patients with CCI, to ultimately mitigate poor outcomes for family members and those for whom they care.

## Methods

This analysis was part of the study that examined expectations about health and prognosis for individuals with CCI, as well as their sources of distress.^29,30^ This patient-oriented^31,32^ and integrated knowledge translation research^33^ included patient partners, researchers, clinicians, and decision makers as equal research team members. We used qualitative and interpretive description; an applied qualitative approach amenable to generating experiential evidence relevant to practice disciplines and clinical applications.^34^ Approval for this research was granted by The University of British Columbia and Fraser Health research ethics boards. Additional methodological details are published elsewhere.^29,30,35^

### Setting and study participants

This research was conducted in a long-term care facility with a 22-bed specialized unit capable of caring for individuals with CCI requiring mechanical ventilation, in British Columbia, Canada, where there is a public healthcare system. We invited all residents with CCI and a family member designated by the resident to participate in the study. Inclusion criteria for the residents with CCI included being cognitively intact (as indicated by the attending physician), ventilator dependent but able to communicate verbally, by mouthing words, or with adaptive technology, and English speaking. We also recruited family members of residents who were not cognitively intact or whose communication could not be understood by the research team. Family members were included who spoke English and were identified by a resident and/or were the designated surrogate decision maker. We invited all the 45 HCPs employed on the unit to participate.

### Data collection

Two team members (AFH and SC), nurses with extensive qualitative interviewing experience, conducted semi-structured, in-person or telephone interviews with family members and residents that lasted 45 to 120 min. We conducted these interviews either independently or in conjunction with a family member (a dyad interview) depending on resident preferences. Our interview guide, published elsewhere,^29^ queried family and resident sources of distress, and expectations of health and disease prognosis. Because we encouraged the participants to communicate the experiences they considered important, both family and resident participants commonly discussed family involvement in resident care. A research nurse with extensive qualitative interview experience conducted telephone interviews, lasting 30 to 45 min, with HCPs rather than in-person at the facility to maintain participant confidentiality. All interviews were audio-recorded, transcribed verbatim, and de-identified.

### Data analysis

Our analysis involved the development of an initial coding frame based on open coding techniques that highlighted transcript segments indicative of emerging patterns, diversities and examples of family, resident, and HCP accounts.^34,36^ We revised the coding frame based on research team deliberations and proceeded to code all interview data using the data management software, NVivo^TM^ version 10. We then used constant comparative methods to group and regroup analytic codes into broader categories and eventual themes, reflecting higher levels of conceptualization.^36^ This involved centering the analysis on family and resident perspectives but then deepening the analysis through comparisons with the HCP data. This enhanced the description and interpretation of family involvement.

## Results

A total of 38 individuals participated: 11 family members, 6 residents with CCI, and 21 HCPs. The 11 family members and 6 residents represented a total of 12 individuals with CCI who resided in the facility (7 family members completed an interview independently, 4 family members completed a dyad interview with a resident, and 2 residents completed an interview independently). See Table 1 for participant information.

**Table 1.** Study participant characteristics.

Family members who participated in an interview	n = 11
Age, years	
20–39	1
40–59	8
60 +	2
Sex	
Male	2
Female	9
Relationship to resident	
Parent	2
Spouse	2
Child	5
Sibling	2
Resident characteristics represented by both resident and family participants	n = 12
Age, years	
20–39	2
40–59	4
60 +	6
Sex	
Male	5
Female	7
Cultural background	
Asian	3
Caucasian	7
South Asian	2
Most recent primary intensive care unit admission diagnosis	
Trauma	1
Sepsis	4
Respiratory failure	5
Neurological insult	2
Time from admission to interview (years)	
<1	6
1–5	4
>5	2
Advanced care directive	
Cardiopulmonary resuscitation	7
Do not resuscitate	5
Method of communication	
Verbal	6
Mouthing words	3
Blinking and nodding	2
Eyegaze technology	1
Level of function	
Wheelchair, independent	4
Wheelchair, dependent	7
Bedridden	1
Healthcare provider participants	n = 21
Position	
Nurse	16
Other healthcare provider	2
Administrative healthcare provider	3
	Mean
Length of employment (years)	6.42
Age (years)	32

The family member, resident, and HCP narratives suggested that family involvement in resident care was multifaceted and entailed family: (1) reorienting their life despite the stress and emotional toll; (2) assuming responsibility for meaningful activities and management of practical matters, yet struggling alone; (3) advocating for care by being present, reminding and pushing, and picking their battles; and (4) figuring out how to contribute to nursing care, but with unclear expectations. Many family members experienced distress and guilt in being unable to provide the care the resident required, which appeared to contribute to these themes. While HCPs echoed the perspectives of the family members and residents in terms of the four main themes, HCPs also offered additional perspectives and insights that are described separately in each theme.

### Family involvement in care entailed reorienting their life despite the stress and emotional toll

Many family members described reorienting their life when their loved one became critically ill, which they continued to do when the resident was transferred to the facility. Some described “uprooting their lives” by moving closer to the facility, changing employment, and switching daily or weekly routines. Most family members expressed love and commitment to the resident and considered their role essential for the resident's wellbeing. They spent substantial time with the resident; some visited the facility for hours on most days while others visited regularly throughout the week. For these family members, attending to the resident's needs was all-consuming. They expressed feeling that “it's all on our shoulders*”* because, other than staff, they were the sole individual involved in the resident's life—a sentiment echoed by the residents.

Juggling their informal caregiver role and attempting to bring the resident some degree of happiness, while also fulfilling home and work responsibilities, left family members exhausted and frustrated. A subset expressed a desire for greater involvement with the resident but competing demands stymied their ability to help, leaving them feeling guilty and sad knowing their loved one was suffering alone.

She's [resident] very depressed… I’m juggling so many balls that I’m only able to come so many times. My dad is working out of town and he can only come so often. So, between the two of us, she gets a visitor probably I’d say on average once a month, maybe twice if she's lucky. So, she's very lonely. (Family Participant 5)

The emotional ups and downs that family members endured as they witnessed the resident's physical and psychological challenges were exacerbated when the resident experienced an episode of acute illness. Several family members conveyed fear of their loved one dying even though they understood the resident's poor prognosis; they vacillated between fear of losing their loved one and fear that their loved one was enduring unnecessary suffering. In reaction to the fear of death, some family engaged in constant vigilance for signs of decline and desired to be ever-present, especially when the resident was unwell.

Despite their emotional distress, the family members commonly described the need to “stay strong” or “be the rock” for the resident. They tried to protect their loved one, careful not to burden the resident, and hid their own emotions. In a dyad interview, the family member was asked about their response when the resident becomes ill, to which they replied: “I just have to stay strong and be there for her. I squash down all the emotions and just soldier on because I have to” (Family Participant 4). Then the resident replied: “Yeah, you hide it” (Resident Participant 4). Several residents commented on their family's attempts to downplay the emotional burden they shouldered. Over time, some family members learned how to cope with the stress of the resident's cycling patterns of acute illness combined with their own life responsibilities. Yet, for other family members, the exhaustion and worry were cumulative leading some to reduce their involvement and time commitment to the resident, as one family member described:

He [brother] finds it very, very stressful. He has a real tough time coming in. I do better than him as far as that goes, but we’ve actually toned it down a little bit the last three or four months. I think we’re only coming in each twice a week, probably an hour and a half at a time instead of three. (Family Participant 7)

#### HCP's cognizant of the stress and emotional toll on family who reoriented their life

The HCPs described a range of family involvement in resident life and care. Some of the HCPs conveyed admiration of the “remarkable” family members who reoriented their lives to be ever-present. Yet, HCPs also expressed concern that some family members were overly committed, preventing them from living a meaningful life apart from the resident. Further, HCPs were cognizant of the burden on some family members who assumed family caregiver responsibilities at the facility, all the while attempting to fulfill multiple life commitments. The pressure on some family members and the emotional toll were described by one HCP:

Most of them [family] also have a busy family life on their own. For some of them you will see tears. They have small kids, they have to look after their care, and then their loved one is here. They [family] have to come here. (HCP Participant 5)

This HCP went on to describe situations where the stress became too much for some family members, particularly when the resident experienced an acute illness episode. Several HCPs highlighted the need for emotional supports and resources for family members. Contrary to these accounts, the HCPs commented on family who were not involved in the lives or care of the resident, with some residents rarely receiving a family visit.

### Family involvement in care entailed assuming responsibility for meaningful activities and management of practical matters, yet struggling alone

Aware of the resident's loneliness, sadness, and boredom, the family members felt compelled to provide companionship and enrich the resident's life. Family members desperately wanted residents to have meaningful interactions or activities in their day and assumed responsibility because: “If I’m not here, he doesn't do anything.” They encouraged new interests, helped with setting goals, and generated ideas or activities to bolster the resident's “will to live.”

The family members and residents described the family as responsible for taking the resident on any sort of outing, or even pushing them around the facility in a wheelchair because staff were always busy with medical tasks and “*run ragged*.” Any activity or outing required the family organize, coordinate, and plan all the details, yet they struggled with the responsibilities given the complex care needs of residents. Several family members and residents recounted terrifying incidents during a resident outing wherein equipment malfunctioned, or misunderstandings posed grave situations. A family member talked about the stress of dealing with potentially fatal mistakes alone:

I take her out, couple of times a year. But it's terrifying every time… Cause that has happened, where the vent has become unplugged and, I didn't realize that the vent had become unplugged until she started turning blue. But it was because the environment was so loud, I could not hear the beeping of the machine, right? So, it's a very fine line, if we’re out too long and her machine dies, she has no breath. (Family Participant 5)

Some family members with resources described figuring out a routine and system for providing the resident with outings. Yet, for others the pragmatic challenges were insurmountable and eventually they gave up trying to plan outings for the resident. In these instances, family commentaries conveyed sadness and guilt knowing that this would result in the resident being confined to the facility.

Residents lived with such fragile health and severe limitations that many of their non-medical needs also became the responsibility of the family. The residents’ finances, appointments, laundry, and belongings such as hearing aids, shavers, and personal items often demanded family involvement. Sometimes these matters were small things, such as replacing lost hearing aids, which nonetheless negatively impacted the resident's daily life; or at times these tasks were major financial arrangements. A family member elaborated on finalizing a real estate inheritance on behalf of the resident: “*Well, now she's financially okay because I sold the apartment… so, now she's okay…*” (Family Participant 8). In sum, not only did family members live with the distress and uncertainty of their loved one's illness, but they also assumed responsibility for the residents’ social support, the planning and logistics necessary for any leisure outings, and the practical management of residents’ lives.

#### HCP's echoed perspectives that responsibility for meaningful activities and practical matters ultimately lay with family

Without the requisite time or resources, the HCPs concurred with the family members and residents that the responsibility to engage residents in meaningful activities, including outings, as well as provide instrumental support, largely fell to family members. Some HCPs believed anything other than “basic care,” referring to life-sustaining routine care of the body, was beyond their scope and they had limited capacity for individualized care.

You’re going to be doing the same thing for all the other residents right? Because, it becomes a special routine if you don't. The way I look at it is we provide basic care for them [residents]. Anything above and beyond that should involve the family. (HCP Participant 3)

Other HCPs described their efforts to provide personalized care and instrumental support, such as specific grooming, taking care of hearing aids or glasses, or keeping track of the residents’ personal effects, yet this was framed as extra care, wherein the responsibility ultimately lay with the family. These HCPs highlighted the unrealistic expectations placed on family members that resulted in family anger and frustration.

### Family involvement in care entailed advocating for care by being present, reminding and pushing, and picking their battles

Most of the family members described making substantial efforts to ensure the resident received good care from staff. These acts of advocacy were ongoing and included the family participants being present, reminding and pushing, and picking their battles.

#### Being present

The family members viewed their presence as necessary to ensure the resident was safe. When visiting, they were always watching and “keeping an eye on things.” For some, this was largely driven by fear and worry that their loved one would not receive adequate care from extremely busy HCPs. The family and resident commentaries revealed a lack of trust in staff at times; therefore, family believed their presence was needed to monitor the care of the resident. A family member who spent full days at the facility recounted how she arrived and found the resident in a wheelchair without all the important supports:

When I came, they had him in the wheelchair. There was no no call bell. And so, for those 20 min or whatever, if something had happened, there was no one—like, they come in once every hour. I just sit here and I—I feel I have to be here, right? (Family Participant 12)

For other family members and residents, the distrust was not connected to real incidents but more a sense of worry and foreboding. A family member shared their concern: “That's my biggest fear, that he's not going to be comfortable. Like something's going to happen” (Family Participant 9).

#### Reminding and pushing

According to the family members and residents, family members understood the unique and specific physical, emotional, and communication needs of the resident in ways the staff were not always aware. Family also described their depth of understanding of resident-specific ways to minimize harm; for instance, in repositioning a particular way to prevent pain. As a result, family felt compelled to be on site to direct care, remind staff of preferences, and advocate for the resident's needs. Several family members acted as a spokesperson for the resident, either because the resident was unable to communicate or preferred not to make requests, or because they perceived it unreasonable to expect staff to recall all the residents’ unique preferences. Yet, they also described having to push or chase down extremely busy staff at times to obtain medical information. Although the families and residents viewed many staff as allies who willingly addressed concerns or ensured processes were in place, the interviews also portrayed a stressed and inflexible care system to which family and staff were forced to bend. A family member described commonly bumping up against a rigid institutional schedule.

There's some [staff] that are really good and friendly… we have a lot of fun. Whereas some just come and do their job, and put him in the chair… I say, ‘Oh, you know, we’d like to do this today.’ ‘Well, you can't. I have this and this and this to do,’ right? Like, —we have to adjust to their schedule. (Family Participant 12)

As a result, family felt caught between wanting to advocate for the resident while also being careful not to alienate or offend staff who were already juggling multiple competing demands.

#### Picking their battles

To advocate for the resident without creating additional problems, the family members assessed what aspects of care were most important and what they might give up. Even though family tempered their advocacy by acknowledging the context of other residents and the busy staff, they also vacillated between giving up or becoming more strategic. A family member described the situation saying, “it's about picking the battle you want to fight, as compared to, almost having a, a sense of resignation sometimes… This isn't going to change” (Family Participant 3). When requests for care were not met, some family saw themselves as the advocate who could escalate an issue to senior HCPs or managers. Other family members were less confident in taking grievances forward, recognized the limitations facing staff, and appeared resigned to the system.

#### HCPs perceived family fear and frustration as driving conflict and disagreement with staff

All HCPs commented on conflicts and disagreements between family members and staff. Several HCPs perceived family members as fearful that the resident cannot communicate their needs, and that they might not receive necessary assistance or good care, driving the family members to feel the need to be present. HCPs also described family members as pushing for care, though this was not framed as advocating for the resident. Rather, most of the HCPs conveyed family members as “demanding,” “complaining,” “interfering,” or “making a fuss” in their “desperate” attempts to care of the resident. The HCPs further depicted family members as frustrated and angry, and “lashing out” when the care provided was not timely or did not meet their high standards. Owing to the competing demands on staff who were caring for multiple complex residents, HCPs considered the difficult position some family were in where they felt the need to ensure their loved one's care was prioritized. Like the family members and residents, HCPs portrayed an inflexible care system, constrained by resources. Yet, they also commented on the different priorities of staff and family members as contributing to family and staff frustration and conflict.

Some family members are actually really, really helpful. They’ll be here like every day. They will help us to do their care. We see other family members like I know how they feel. They want their own resident to be cared for first. But then also we have our own routine. We can't just go if it's not like a priority, or if it's nothing like an emergency. Then, every time we have to explain, okay so this person is more of a priority on us. (HCP Participant 16)

### Family involvement in care entailed figuring out how to contribute to nursing care, but with unclear expectations

Many of the family members were active in providing nursing care because they perceived it as their duty, they wanted to contribute to quality care for the resident, and they felt a sense of guilt when not involved. Family members performed a range of tasks including repositioning residents, massage, personal grooming, range of motion exercises, toileting, suctioning, and managing enteral nutrition equipment. Further, every family member and resident commented on the impression of hectic, time-pressured staff; therefore, they saw family involvement as necessary. Because staff were so busy, family learned to adjust their expectations, fit into established routines, and supplement nursing care, as evident in one family member's example:

A lot of times they’ve [staff] told me, like, he's asked for a reposition. ‘Well, we have to go finish our rounds or do our reports,’ and then they’ll come back half an hour or an hour later… —that's why I’ll pull the wedge out or I’ll put the pillows in. I try to make him comfortable until they show up. (Family Participant 10)

In many instances the family members described figuring out how to perform nursing tasks by observing staff carefully, and occasionally the staff provided them with training, for which they were grateful. However, the family members also conveyed uncertainty, ambiguity, and unease being responsible for some of these tasks. Though many family members were willing to step in and provide care, taking pride in their contributions, some were unclear about staff expectations. The lack of institutional parameters around participation in nursing care left some family feeling ill prepared and overwhelmed. Family involvement was often encouraged and appreciated by staff, but there were situations when family involvement seemed limited, which sent mixed signals. A family member who usually helped position their mother for the night and assist with evening care mentioned that sometimes they were told by staff to wait outside the room until they finished. This was confusing for the family member because they perceived their involvement as a sign of intimacy and caring for the loved one. On the other hand, both family and resident participants recounted situations when staff seemed reliant on family members’ contributions to care.

#### HCP's considered family involvement in nursing care essential

Most HCPs considered the family as members of the care team, and shared examples of family members performing personal care, assisting with mealtime, physiotherapy, and “simple medical care” such as physical repositioning or oral suctioning. HCPs framed family involvement as essential for “resident-oriented care,” acknowledging the insight family members had into the unique and specific needs of the resident, especially when first admitted to the facility. The HCPs also highlighted their inability to meet all the residents’ needs simultaneously, and the benefit of family taking on some of the nursing care, as explained by one HCP:

For family members, simple techniques such as a mouth suctioning. Or like just a simple thing. Because sometimes when everything, like vents are beeping, we can't just attend them… Like mouth suction it's something that is needed. But I mean the family member can do it right? Like let's say if I’m busy, I can't go there right away, but they need a mouth suction which regular family can even do. (HCP Participant 11)

Like other participants, this HPC went on to describe the need for education and training to enable family to provide safe care. In contrast to family members and residents, HCPs suggested that there were also family members who were unwilling to be involved in care, who declined requests from staff to assist them.

## Discussion

In this qualitative study of family involvement in the life and care of a resident with CCI, the burden of caregiving was significant. This contrasts with the assumption that family members are relieved of their caregiver responsibilities when the patient with CCI is in a medical facility rather than requiring care at home.^37^ Our study illustrates the extensive and complex roles and responsibilities that family members of individuals with CCI assume and how these then contribute to the psychological distress, feelings of being emotionally overwhelmed, exhaustion, uncertainty, and even conflict reported by others.^17,19^ These findings are noteworthy considering that in other illness contexts, high levels of caregiver burden^38^ have been found to contribute to poor mental and physical health,^39^ and even caregiver morbidity and mortality risk.^40,41^ Of note, Dale et al.,^12^ found that family caregivers of individuals requiring prolonged mechanical ventilation in a specialized weaning unit reported stress-related health changes including alterations in eating habits, body weight, sleep quantity and quality, and energy levels. Other researchers^42^ have described the cumulative effects of prolonged stress for family members of those with CCI, which, in our study, was unsustainable for some, who eventually reduced their involvement. Efforts to prepare family members, not just for the poor prognosis of the patient but the enormity of the possibility of caregiving and caregiver burden are warranted.

Our study explicates some of the ways in which family members of individuals with CCI advocate for their loved one, including being ever-present and reminding and pushing staff. This research aligns with others who have suggested that family of individuals with CCI or prolonged mechanical ventilation can be motivated by the fear of poor patient care and outcomes to safeguard their loved one.^12,43^ In safeguarding, they seek to protect the patient from harm, which in the ICU can be expressed as extended bedside presence for example. According to Davidson et al.,^43^ this signals a heightened emotional state and the need for empathic support. Emotional distress was common among the family members in our research and recognized by staff who emphasized the need for family supports. Going forward, educational programs tailored to family of CCI individuals are perhaps warranted, along with robust psychological support.^44^ Research in the ICU suggests that family presence is necessary for family engagement, which when paired with an educational program, can improve patient and family outcomes.^43^

Patient-centred and family-centred care has gained traction in the ICU with increasing evidence that this model of care can improve family satisfaction, patient experiences, medical goal achievement, and family mental health outcomes.^45^ Based on our research findings, family-centred care, an approach to healthcare that is respectful of and responsive to individual families’ needs and values,^43^ is particularly relevant in the context of chronic critical illness and research is needed to develop and evaluate interventions. The specifics of these interventions have yet to be identified. For example, a recent randomized clinical trial that included the provision of information and at least two family meetings led by palliative care specialists, did not reduce anxiety or depression in family surrogate decision makers of patients with CCI.^46^ We concur with White^47^ that low-dose interventions may be inadequate to improve family outcomes. We also contend that a broader conceptualization of family involvement in care that extends beyond surrogate decision maker would more accurately reflect the reality of family caregiving. Efforts to facilitate family engagement more broadly might be effective. For example, structured, hands-on training for family desiring involvement in medical care, coupled with guidance for staff about how to engage family safely and effectively, might be one solution.

### Strengths and limitations

The strength of this qualitative research lies in the information power, which was enhanced through the high quality of the in-depth interviews, and characteristics of study participants being highly specific for the study purpose.^48^ A further strength was the inclusion of dyadic interviews, which helped to generate particularly relevant insights from the recipients of family care. Dyadic interviews facilitated resident participation when the family member functioned as a communication partner or a source of support when the interview elicited strong emotions.^35^ A study limitation was the inclusion of participants who were willing and able to be interviewed. With this self-selection, particularly of family and resident participants, the full spectrum of family experiences of involvement in care was not elucidated. To this point, HCP accounts suggested that there are family members who are only minimally involved, if at all, in resident care. Characteristic of qualitative research, our findings provide insights about what family involvement in the care of a person with CCI in long-term care entails that might be relevant to other care environments, such as long-term acute care hospitals and rehabilitation facilities, but we do not claim that they are generalizable. Study participants were all from one facility designed to provide specialized care in a publicly funded healthcare system, which might differ from other care contexts.

## Conclusion

In this qualitative study, family member involvement in the care of a person with CCI in long-term care was complex and multifaceted. Family members commonly reoriented their life, assumed responsibility for meaningful activities and management of practical matters, and advocated for and contributed to medical care. Strategies to address unmet family needs specific to the roles and responsibilities that family assume remain unknown but could potentially enhance the ability of family to be meaningfully involved in the resident's life as well as improve family outcomes.
